# New alkyl-phosphate bonded stationary phases for liquid chromatographic separation of biologically active compounds

**DOI:** 10.1007/s00216-012-6134-0

**Published:** 2012-06-22

**Authors:** Szymon Bocian, Alicja Nowaczyk, Boguslaw Buszewski

**Affiliations:** 1 Department of Environmental Chemistry and Bioanalysis, Faculty of Chemistry, Nicolaus Copernicus University, Gagarin 7, 87-100 Torun, Poland; 2 Department of Organic Chemistry, Faculty of Pharmacy, Collegium Medicum in Bydgoszcz, Nicolaus Copernicus University, Jurasza 2, 85-094 Bydgoszcz, Poland

**Keywords:** Alkyl-phosphate bonded phase, Immobilized artificial membrane, Liquid chromatography

## Abstract

A new type of bonded stationary phase for liquid chromatography, with the properties of immobilized artificial membranes, has been synthesized. Alkyl-phosphate adsorbents were obtained by modification of aminopropyl silica gel. The structures of the synthesized materials were confirmed by use of instrumental techniques—elemental analysis, infrared spectroscopy (FTIR), and ^13^ C and ^29^Si CP/MAS NMR. Analysis revealed that the adsorbents mimic the phospholipids present in natural cell membranes. The new synthesized alkyl-phosphate stationary phases may be used for liquid chromatographic separation of biologically active compounds of different polarity.

## Introduction

In 1989, Pidgeon et al. [1] first described a new type of bonded stationary phase for liquid chromatography. That bonded phase simulated the phospholipid bilayer of cell membranes. It consisted of a phospholipid analog monolayer covalently bonded to the silica surface and was called an *immobilized artificial membrane* (IAM) [2, 3]. The IAM bonded phase more closely mimics the interaction of analytes with biological membranes rather than the classical octadecyl stationary phase. The is because of combinations of possible hydrophobic, ion pairing, and hydrogen bonding [4, 5].

The IAM phases were prepared by immobilizing a single chain containing a phosphocholine headgroup. Other types of IAM phases contain a long-chain alcohol with polar OH groups protruding from the surface or a long-chain fatty acid containing OCH_3_ groups [6].

A different group of non-standard stationary phases for liquid chromatography are the *N*-acylamide adsorbents (AP) [7]. These materials contain an amide bond formed by reaction of a primary amine ligand with a long-chain carboxylic acid. With AP stationary phases one can observe types of molecular interaction slightly different from those of octadecyl C_18_ bonded phases [8].

Another type of stationary phase which tries to simulate natural membranes is the cholesterol bonded phase. These adsorbents may be synthesized in several ways. The first attempt was described by Siouffi [9]. The method relies on reaction of the terminal amine of bonded aminopropyl silica with cholesteryl chloroformate. The cholesterol molecule in this stationary phase is bonded by use of a short linkage. Another method was published by Pesek et al. [10], who used cholesteryl undecanoate bonded to silica via hydride formation and hydrosilylation. He obtained a bonded stationary phase in which the cholesterol molecule was immobilized by use of a relatively long linkage. The advantage of this method seems to be the presence of a long hydrocarbonaceous spacer arm with high mobility. At the same time Buszewski et al. [11], independently reported a method for synthesis of a cholesterol bonded phase by modification of an aminopropyl intermediate.

The cholesterol bonded phase is a unique separation material the analytical capabilities of which are only beginning to be investigated [5, 12–14]. These materials can be successfully used to separate mixtures by both reversed-phase and normal-phase chromatography [8, 13, 15–17]. The cholesterol bonded phases have high resolving power for some samples, which may be attributed to their liquid crystal properties [9, 18]. One advantage of these packing materials is the possibility of using them with highly aqueous mobile phases without any evidence of collapse of the bonded phase. This drastically reduces solute retention [12].

Our objective in this work was to synthesize a new generation of alkyl-phosphate bonded stationary phases for liquid chromatography with properties similar to those of the cell membrane. On the basis of previous experience with the synthesis of cholesterol and *N*-acylamide adsorbents, the stationary phase prepared in this work was obtained by chemical modification of aminopropyl silica gel. The structure of the chemically bonded ligand was confirmed by elemental analysis, and solid state NMR and FT-IR spectroscopy. Potential chromatographic application of the materials is also presented.

## Experimental

### Materials

The solid support of in-house-made phases was Kromasil 100 porous spherical silica gel (Akzo Nobel, Bohus, Sweden) with particle diameter 5 μm. The mean pore diameter of this material is 100 Å and specific surface area is 313 m^2^ g^−1^.

Synthesized materials were compared with the IAM.PC bonded stationary phase (Regis Technologies, Morton Grove, IL, USA).

### Reagents

Aminopropyltrimethoxysilane, morpholine, phosphoryl chloride, decanol, and octadecanol were purchased from Alfa Aesar (Karlsruhe, Germany). Methanol, toluene, chloroform, and hexane (all analytical-grade) and methanol for HPLC analysis (“gradient”-grade) were obtained from J.T. Baker (Deventer, The Netherlands).

### Apparatus

The liquid chromatograph was an HP Model 1050 (Hewlett Packard, Waldbronn, Germany) equipped with a four-channel gradient pump, an autosampler with a 100-μL loop, a diode-array detector, and a data acquisition station (ChemStation software).

The degree of coverage of the surface by alkylsilyl ligands (*α*_RP_) was calculated on basis of the carbon percentage determined by use of a Model 240 CHN analyzer (Perkin Elmer, Norwalk, USA).

FTIR spectra in the range *ν* = 4,000–400 cm^−1^ were recorded on a Spectrum 2000 instrument (Perkin–Elmer Norwalk, USA).

Solid state NMR spectroscopy was performed with a Bruker (Karlsruhe, Germany) MSL 300 spectrometer, with samples of 200–300 mg in double bearing rotors of ZrO_2_. ^29^Si CP/MAS NMR spectra were recorded with a pulse length of 5 μs, a contact time of 5 ms, and a 2-s pulse repetition time. For ^13^ C CP/MAS NMR the pulse length was 5 μs, contact time 3 ms, and pulse repetition time 2 s. All spectra were externally referenced with liquid tetramethylsilane (TMS) and chemical shifts (*δ*) are given in parts per million (ppm).

Adsorbents were packed into 125 mm × 4.6 mm i.d. stainless steel columns, by use of the slurry method, by means of laboratory-made equipment and a Haskel (Burbank, CA, USA) packing pump. Approximately 1.5 g modified silica was made into the slurry with 15 mL chloroform and placed in the packing apparatus. Methanol was used as a packing pressurizing solvent during the filling process. Columns were packed at a constant pressure of 40 MPa.

### Synthetic procedure

Before chemical modification of the bare silica gel, a sample of the adsorbent was placed in a glass reactor specially designed to protect the reagents from contact with the external environment. Silica gel was treated at 180 °C under vacuum (10^−2^ Pa) for 10 h to remove physically adsorbed water. The temperature was then reduced to 120 °C and γ-aminopropyltrimethoxysilane was added. Silica support surface modification with aminopropyl ligands was performed under solvent-free conditions as described in detail in Refs. [13, 15].

After 12 h, the product of the reaction was washed with toluene, methanol, and hexane, and dried. The aminopropyl silica was then placed in a glass reactor and heated to 100 °C.

**Fig. 1:**
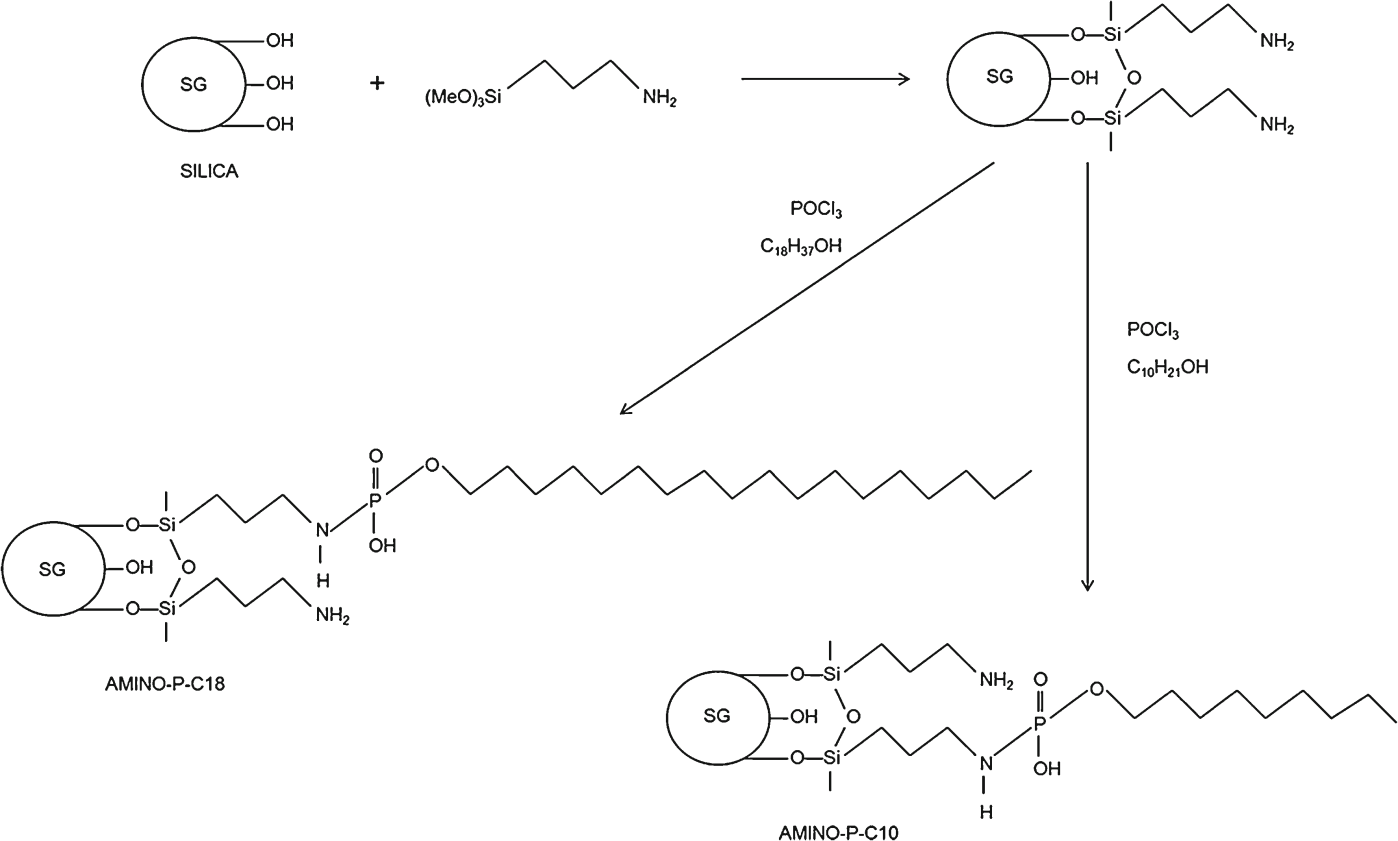
Schematic diagram of the procedure for synthesis of the stationary phases

The aminopropyl silica was then modified to obtain the new chromatographic materials—Amino-P-C10 and Amino-P-C18 containing 10 and 18 carbon atoms, respectively, in the main chains (Fig. 1). Modification was conducted in dry toluene. The reactions were performed for 12 h, and the final products were washed as described above. The adsorbents obtained contain alkyl chains connected to aminopropyl ligands by use of phosphate groups (Fig. 1).

## Results and discussion

### Elemental analysis and coverage density

The synthesized adsorbents were submitted for elemental analysis. Results from chemical modification of the silica gel surface, i.e. carbon, nitrogen, and hydrogen content after each bonding reaction determined by elemental analysis, are presented in Table 1. These results enable precise calculation of the coverage density (*α*_RP_) of the silica surface by use of the Berendsen equation [19]:

**Table 1:** Physicochemical properties of the alkyl-phosphate stationary phases

Property	Abbreviation	Amino	Amino-P-C10	Amino-P-C18	IAM.PC
Carbon content (%)	P_C_	2.83	8.43	9.33	5.2^a^
Nitrogen content (%)	P_N_	1.25	0.91	1.15	0.4^a^
Hydrogen content (%)	P_H_	1.31	1.94	2.20	–
Coverage density (μmol m^−2^)	α_RP_	2.80	1.64	1.04	–

^a^Data from producer

1 $$ {{\alpha }_{{RP}}}^{I} = \frac{{{{{10}}^{6}}{{P}_{C}}}}{{1200{{n}_{C}} - {{P}_{C}}\left( {{{M}_{1}} - {{n}_{X}}} \right)}}\cdot \frac{1}{{{{S}_{{BET}}}}}\:\:\:\left( {\mu {\text{mol}}/{{{\text{m}}}^{{ - 2}}}} \right) $$

where *α*_RP_ is the coverage density (μmol m^−2^), *P*_C_ is the percentage of carbon (%), *n*_C_ is the number of carbon atoms in the ligand, *M*_1_ is the molar mass of the ligand, *n*_x_ is the number of functional groups in the reactive group of the silane, and *S*_BET_ is the specific surface area (m^2^ g^−1^).

The coverage density of amino and phospho-alkyl ligands of both materials is listed in Table 1. All of the synthesized adsorbents have the same coverage with aminopropyl ligands. Higher ligand concentrations after the second step of the reaction were observed for the Amino-P-C10 phase than for Amino-PC18. It was probably because of steric hindrance by the longer 18-carbon-atom chains. For Amino-P-C10, more than half of the amino groups are modified with phospho-alkyl ligands. Lower coverage density of alkyl ligands in comparison with commercially available octyl or octadecyl bonded phases enables participation of phosphate groups in the retention. With higher coverage density deeply located phosphate groups may be shielded and not interact during chromatographic elution. The coverage density of alkyl ligands may be controlled during synthesis by changing the composition of the reagent mixture.

### NMR investigations

**Fig. 2:**
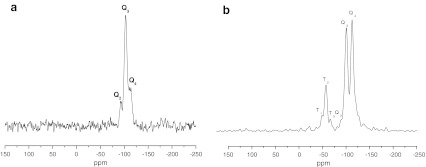
^29^Si NMR spectra of bare silica gel (**a**) and silica modified with aminopropyl ligands (**b**)

The amino, Amino-P-C10, and Amino-P-C18 bonded phases were investigated by use of ^13^ C and ^29^Si CP/MAS NMR measurements. Both spectroscopic techniques are very powerful methods for characterizing chemically bonded organoligands of stationary phases. Analysis of ^29^Si CP/MAS NMR spectra obtained for unmodified silica gel revealed the presence three types of silanol on the silica surface, i.e. geminal silanols (Q_2_; *δ* = −92 ppm), single silanols (Q_3_; *δ* = −102 ppm), and siloxane groups (Q_4_; *δ* = −112 ppm). The intensity of the Q_2_ and Q_3_ signals decreases after derivatization whereas that of Q_4_ increases as a result of siloxane bond creation (Fig. 2). After the modification reaction new signals are observed. Their chemical shifts depend on the functionality of the silanes used, and appeared at T_3_ (*δ* = −66*.*3 ppm) and T_2_ (*δ* = −59*.*2 ppm). The absence of T_1_ signals at *δ* = −48 ppm is indicative of high crosslinking of the ligands on the silica surface [20–24].

**Fig. 3:**
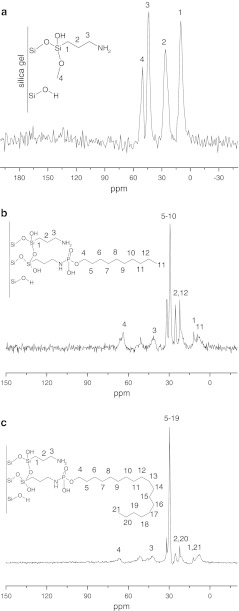
^13^C NMR spectra of amino (**a**), Amino-P-C10 (**b**), and Amino-P-C18 (**c**) bonded phases

The ^13^ C CP/MAS NMR spectra obtained for the stationary phase (Fig. 3) confirmed the presence of alkyl bonded ligands. In the range of chemical shift values from +12 to +35 ppm, peaks corresponding to the bonded organic moiety can be observed [25–29]. These signals increased substantially with the length of the main chain from the Amino-P-C10 to Amino-P-C18 bonded phases.

On the basis of NMR and FT-IR investigation of the synthesized materials, the structures proposed for the synthesized adsorbents are presented in Fig. 3. On both materials there are unbonded amine groups which affect the hydrophobic and/or polar properties of the packing material.

### FT-IR spectroscopy

**Fig. 4:**
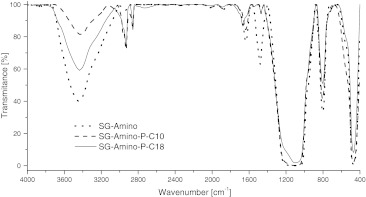
IR spectra of amino, Amino-P-C10, and Amino-P-C18 bonded stationary phases

Each stage of the stationary phase synthesis was documented by means of infrared and nuclear magnetic resonance spectroscopy. Typical FT-IR spectra recorded for the amino, Amino-P-C10 and Amino-P-C18 materials are presented in Fig. 4.

As was easy to predict for the synthesized material, the most prominent peaks are those characteristic of any hydrocarbonaceous bonded ligands at approximately *ν* ≈ 2,900–2,800 cm^−1^ [15]. These signals are indicative of considerable hydrophobicity of the surface covered by the organic stationary phase [13]. At *ν* ≈ 1,100 cm^−1^ the siloxane asymmetric stretching vibrations are observed. In addition, siloxane bending vibrations are visible at *ν* ≈ 800 cm^−1^. The signal at *ν* ≈ 3,300–3,600 corresponds to N–H stretching vibrations in amines and O–H vibrations in hydroxyl groups. The intensity of these signals decreases after modification of the amines, and the signal reduction is higher for the Amino-P-C10 phase as a consequence of the higher surface coverage with alkyl-phosphate ligands.

The characteristic peak at *ν* ≈ 1,535 cm^−1^ is attributed to bending vibrations of amino groups. That peak almost disappeared from the spectra of the alkyl-phosphate phases.

The materials obtained have specific properties connected with the structure of chemically bonded ligands. In the bonded moiety, two zones with different properties—polar and hydrophobic—may be identified. First, starting from the bottom is the silica surface with polar residual silanols. On this surface polar interactions (hydrogen bonds) are predominant. The organic chain of the aminopropyl ligand and the decyl or octadecyl chain are hydrophobic. However, the hydrophobicity of the propyl linkage is not of significant importance. The most important hydrophobic interactions are those of the of C_10_ and C_18_ chains. Amine and phosphate groups are polarized and interact with mobile phase components and solutes via polar interactions. Depending on mobile phase pH, these groups may be ionized. This could result in the presence of charge inside the bonded ligands.

This type of bonded stationary phases may be used in reversed-phase liquid chromatography and/or in HILIC mode depending on the composition of thee mobile phase. This enables separation of hydrophobic molecules and polar compounds with highly organic mobile phases.

### Surface modeling

For better description of the properties of these bonded stationary phases, molecular modeling studies were conducted. A common way of visualizing the distribution of charge in a molecule is to map the electrostatic potential in the form of a 3D plot or a 2D contour plot of the electrostatic potential distribution (MEP). Regions of the electron density surface that are more negative than others in an MEP are colored red. Regions in the MEP that are less negative (or are positive) are blue. The color spectrum indicates the trend in charge from most negative (red) through green and yellow (neutral) to positive (blue) [30, 31].

Taking into account the molecular interaction, it would be more informative to discuss hydrophobicity and hydrophilicity. From this perspective, if a specific area is more red or blue, the more hydrophilic is the molecular fragment. Consequently colors ranging from green to yellow account for the hydrophobic properties of a group.

Our modeling study involves the charge distribution in the studied bonded stationary phases. All calculations were performed using the Gaussian 03 program [32]. The visualizations were prepared by use of the GausView 4.1 [33]. The starting model of the silica gel surface was taken from our previous work [34]. In the next step the aminopropyl, Amino-P-C10, and Amino-P-C18 bonded stationary phases were constructed by substituting the hydrogen atoms from the silanol groups by 1, 2, 3, and 4 aminopropyl and amino phospho-alkyl ligands randomly grafted on to the slice of the silica gel. All the molecules’ geometries were optimized by use of the molecular mechanics method MM3 until the root mean square gradient became smaller than 0.01 kcal mol^−1^ Å^−1^ (Table 2). Single point energy (SPE) calculations were then performed at the DFT/B3LYP level of theory by use of the 6-31 G** basis set. Later, using the surface data generated from Gaussian checkpoint files, and GaussView 4.1 software, the distribution of charge in a molecule was calculated. To obtain a 3D plot of the MEP, the electrostatic potential cube file was calculated from total SCF density. The contour maps of the electrostatic potential were then drawn using a distance between grid points of 0.02 Å and the isovalues 0.0004. All computations were performed on an HP-6200 wx workstation. The Gaussian software suite was used to calculate the electrostatic potential maps and surfaces as the distribution of the potential energy of a unit positive charge in a given molecular space, with a resolution controlled by the grid density.

**Table 2:** Geometry of the alkyl-phosphate stationary phases

Dihedral angles	(°)	Angles	(°)	Bonds	(Å)	(kJ mol^−1^) [36]
Si–O–C–C	180	Si–O–C	120	Si–O	1.62	435
O–C–C–C	180	O–C–C	110	O–C	1.42	345
C–C–N–P	180	O–Si–O	90	C–C	1.52	338
		N–P–O	110	C–N	1.47	290
		O–P–O	120	C–H	1.12	435
				P–N	1.65	305
				H–N	1.11	389
				P–O	1.62	355
				P=O	1.50	605

**Fig. 5:**
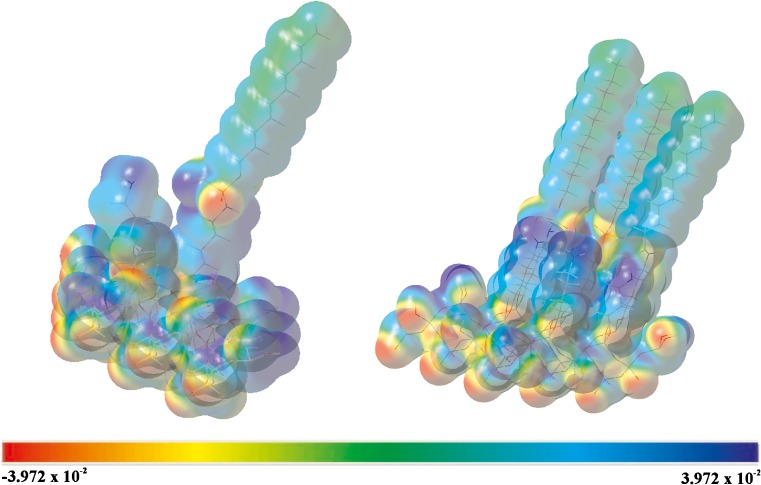
Calculated electrostatic potential maps for Amino-P-C10

According to the basic theory of electrostatics, negative potentials correspond to attraction of the probe unit charge by the higher electron density in space whereas positive potentials correspond to repulsive interactions of the probe charge with unshielded nuclear forces present in the low electron density spaces [35]. Amino-P-C10 MEP plots are presented in Fig. 5. The color code of these maps is in the range between −0.03972 a.u. (deepest red) and 0.03972 a.u. (deepest blue). As can be seen from the MEP of the Amino-P-C10, regions having negative potential are over the electronegative (oxygen) atom and regions having positive potential are over the hydrogen atoms. The calculated MEP shows that the most negative electrostatic potential is concentrated around the oxygen and nitrogen atoms. For nitrogen, the presence of hydrogen (amino group) results in a strongly positive potential at their side, shielding the effects of N atoms alone. Regions having extremely positive electrostatic potential are these including H atoms near the silica gel surface, which implies hydrophilic character. However, around the H atoms of the alkyl chain attached to the phosphate group, electrostatic potential is in the range specific for hydrophobic character. From this result, we can say that the bonded stationary phases have hydrophilic character at the vicinity of the silica surface and hydrophobic properties starting approximately the 6–7 Å above it.

### Chromatography

The synthesized stationary phases were packed into stainless-steel columns. The column void volume was determined by injection of an unretained marker (thiourea) and was 1.66 mL and 1.64 mL for Amino-P-C10 and Amino-P-C18, respectively. Void volumes of the columns were measured using 60 % MeOH in water as a mobile phase.

**Fig. 6:**
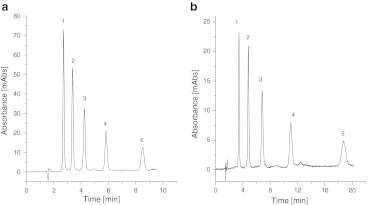
Separation of benzene homologues benzene (*1*), toluene (*2*), ethylbenzene (*3*), propylbenzene (*4*), and butylbenzene (*5*) on Amino-P-C10 (**a**) and Amino-P-C18 (**b**) with 50 % MeOH in water as a mobile phase

Both stationary phases were tested by reversed-phase liquid chromatographic separation of alkylbenzenes. The results are presented in Fig. 6. On both packing materials the mixture was separated by use of 50 % MeOH in water as mobile phase. The columns tested furnished peaks with very good asymmetry factor (*f*_AS_) from 0.87 to 1.1. These stationary phase may therefore be applied to separation of hydrophobic compounds by reversed-phase liquid chromatography.

Retention all of the compounds tested was higher on the Amino-P-C18 bonded phase. The results presented show this packing material is more hydrophobic than the Amino-P-C10 adsorbent. This phenomena can be explained by the greater hydrophobicity of the C_18_ ligands, despite the lower coverage density of C_18_ chains in the comparison with the shorter ones. The carbon loading of the Amino-P-C18 material (9.33 %) is slightly higher that of Amino-P-C18 (8.43 %). This results in greater hydrophobicity. An analogous situation is observed for traditional reversed-phase packings—C_18_ bonded phases are more hydrophobic than C_8_, irrespective of the usually higher coverage density of octyl adsorbents [11, 36].

**Fig. 7:**
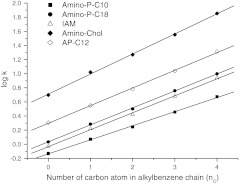
Changes of the retention (log *k*) with number of carbon atoms in the alkylbenzene chain

As seen in Fig. 7 the retention of alkylbenzenes increases linearly with number of carbon atom in the chain. This confirms the chain–chain interaction governing retention of these hydrophobic compounds.

Retention of alkylbenzenes was compared with data obtained for IAM, AP-C12, and Amino-Cholesterol stationary phases, which also mimic the structure of cell membranes. Synthesized alkyl-phosphate adsorbents have a structure similar to that of the IAM phase. Thus, the most similar results were obtained on IAM and Amino-P-C18. In Fig. 7 a similar trend in retention with increasing number of carbon atoms in the solutes is observed. This confirms the effect of long alkyl chains on retention of the alkylbenzenes on both phases. The slope for the more hydrophobic Amino-P-C18 phase is steeper than that for Amino-P-10, which corresponds to the better selectivity of the former for alkylbenzenes.

Comparison of the Amino-P-C10 and Amino-P-C18 materials with Amino-Cholesterol adsorbent shows that alkyl-phosphate phases have much lower hydrophobicity. Retention of alkylbenzenes on the Amino-Cholesterol phase is much stronger which distinguishes it from the IAM and alkyl-phosphate adsorbents.

The hydrophobic/polar properties of the AP adsorbent are between those of the alkyl-phosphate and amino-cholesterol stationary phases. This material has strong hydrophobicity, which is slightly reduced by the presence of amide groups. Despite of the presence of polar groups, this material is most suitable for separation of hydrophobic compounds, rather than polar compounds.

**Fig. 8:**
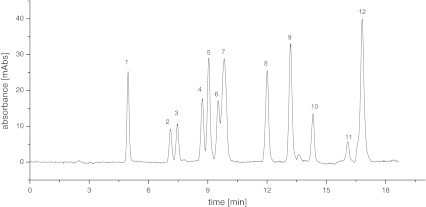
Example of the separation of beta-blockers on Amino-P-C10

The Amino-P-C10 stationary phase, which is less hydrophobic than Amino-P-C18, may be used for separation of more polar compounds. An example of a potential application of Amino-P-C10, chromatographic separation of twelve beta-blockers, is shown in Fig. 8. The sample consisted of atenolol (1), nadolol (2), metoprolol (3), esmolol (4), talinolol (5), oxprenolol (6), acebutolol (7), betaxolol (8), propranolol (9), alprenolol (10), talinlol (11), and nebivolol (12) in order of elution. All of these compounds have polar functional groups in their structures. The mobile phase was MeOH–water in linear gradient mode from pure water to 90 % MeOH in 20 min. Separation of these beta-adrenolytic drugs cannot be achieved with Amino-P-C18 as stationary phase under the same conditions.

The two different cholesterol-bonded stationary phase were also used for separation of beta-blockers. Unfortunately, the high hydrophobicity of these stationary phases [14] makes the separation impossible. The analytes were eluted almost in the void volume of the columns. The results were much worse than for the Amino-P-C10 stationary phase. However, the beta-blockers can be separated on Amino-Cholesterol stationary phases by use of gradient elution with a buffered mobile phase [37]. Use of Amino-P-C10 enables separation without addition of buffer to the mobile phase.

For separation of polar compounds, this column has very good chromatographic properties. The asymmetry factors (*f*_AS_) of the peaks were in the range 0.89–1.29. Two pairs of compounds (4, 5 and 6, 7) were not fully separated. The idea was only to show the potential usage of the synthesized material for chromatographic separations (Table 3)

**Table 3:** Chromatographic data from separation of beta-adrenolytic drugs

Compound	Retention factor (*k*)	Selectivity (*α*)	Number of theoretical plates	Asymmetry factor (*f*_AS_)
1	2.03	–	9,735	1.08
2	3.33	1.64	12,787	0.89
3	3.55	1.07	14,359	1.02
4	4.32	1.22	17,062	1.14
5	4.51	1.04	18,960	0.91
6	4.80	1.06	16,738	1.06
7	4.97	1.03	6,180	0.61
8	6.31	1.27	24,004	0.98
9	7.03	1.11	24,287	0.92
10	7.73	1.10	17,847	1.21
11	8.80	1.14	8,705	1.29
12	9.24	1.05	24,469	0.91

.

**Fig. 9:**
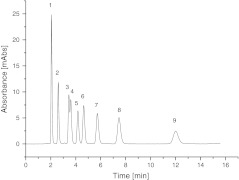
Example of the separation of nucleosides on Amino-P-C18 using pure water as a mobile phase

Another possible use of these new stationary phases is separation of nucleosides and modified nucleosides. In Fig. 9 the separation of nine nucleosides on Amino-P-C18 is presented. This sample consisted of cytidine (1), uridine (2), guanosine (3), 1-methylinosine (4), thymidine (5), 1-methylguanosine (6), N2-methylguanosine (7), adenosine (8), and 1-methyladenosine (9). Separation was achieved by use of pure water as mobile phase. These new stationary phases can be used with pure water as mobile phase without any evidence of stationary phase collapse. Analysis was performed without addition of buffer to the mobile phase. This separation cannot be performed with Amino-P-C10 and a wide range of mobile phase composition. On Amino-P-C10, nucleosides were eluted almost in the dead volume of the column.

The results presented suggest the phosphate groups have a significant effect on the mechanism of retention on alkyl-phosphate phases. The effect of the polar amine residues should not be overlooked. Alkyl-phosphate packings may be used for separation of polar compounds which are very poorly retained on alkyl-bonded stationary phases in reversed-phase chromatography. The presence of the polar amino and phosphate groups in the structures of the bonded phases gives them hydrophilic properties and leads to better solvation in water-rich mobile phases, as was proved for *N*-acylamide bonded stationary phases [38]. Thus the stationary phase is stable when pure water is used as mobile phase. Detailed information about the chromatographic properties and potential uses of these phases will be topics for further investigation.

## Conclusions

A new type of stationary phase for liquid chromatography has been synthesized. The materials obtained were investigated by instrumental analysis. On basis of IR and NMR spectroscopy, in the structure of the bonded moiety long organic chains (C_10_ or C_18_) were attached to aminopropyl silica by use of a phosphate group. The presence of the long ligand and phosphate groups make these stationary phases similar to the phospholipids which constitute the bilayer cell membrane. Molecular modeling investigations showed that the charge distribution in our innovative stationary phases significantly affected their hydrophobic–polar nature. The properties of Amino-P-C18 adsorbent are similar to those of the commercially available IAM phase. However, the much simpler structure of Amino-P-C18 means this material is easier to produce.

The physicochemical properties of these adsorbents enable their use for separation of both hydrophobic and polar substances. Changing the length of main chain over a wider range enabled the preparation of chromatographic materials with different hydrophobic–polar properties.

Application of the adsorbents to the separation of beta-blockers and nucleosides shows their potential application for separation of biologically active compounds with polar properties. The new materials give symmetrical peaks and enable highly selective and efficient separations. An additional advantage of these materials is that they can be used under completely aqueous conditions without collapsing and provide good peak shapes of polar compounds without addition of buffers to the mobile phase.
